# Molecular Characterization of *Clistobothrium* sp. Viable Plerocercoids in Fresh Longfin Inshore Squid (*Doryteuthis pealeii*) and Implications for Cephalopod Inspection

**DOI:** 10.3390/pathogens9070596

**Published:** 2020-07-21

**Authors:** Lisa Guardone, Alice Giusti, Ewa Bilska-Zajac, Renato Malandra, Miroslaw Różycki, Andrea Armani

**Affiliations:** 1 Department of Veterinary Sciences, University of Pisa, 56124 Pisa, Italy; alice.giusti183@outlook.it (A.G.); andrea.armani@unipi.it (A.A.); 2 Department of Parasitology and Invasive Diseases, National Veterinary Research Institute, 24-100 Puławy, Poland; ewa.bilska@piwet.pulawy.pl (E.B.-Z.); mrozycki@piwet.pulawy.pl (M.R.); 3 Wholesale Fish Market of Milan, ASL of Milan, 20137 Milan, Italy; RMalandra@ats-milano.it

**Keywords:** cestode, helminths, cephalopods, food hygiene, defect, visual inspection

## Abstract

Cephalopods, an appreciated seafood product, are common hosts of marine cestodes. The aim of this work is to report visible alive plerocercoids in longfin inshore squid (*Doryteuthis pealeii*), a cephalopod species commercialized as fresh and whole in Italy. Seventy *D. pealeii* from the Northwest Atlantic (FAO area 21) were collected and visually inspected. In total, 18 plerocercoid larvae were found in the viscera of 10 host specimens (P: 14.3% 95% CI 7.1–24.7; MI: 1.8, MA: 0.26; range 1–4) and molecularly analyzed targeting the variable D2 region of the large subunit (LSU) rRNA gene and the cytochrome *c* oxidase subunit I (*COI*) gene. The molecular characterization allowed to identify all the plerocercoids as *Clistobothrium* sp., a cestode of the Phyllobothriidae family with Lamnidae sharks as definitive hosts, and cephalopods as second intermediate hosts. These findings represent the first molecular record of *Clistobothrium* sp. in *D. pealeii*, thus contributing to elucidate its poorly known life cycle. Even if not affecting consumer’s health, these visible parasites may represent a reason for disgust for consumers. Therefore, the results suggest that Food Business Operators should also check for the presence of these visible parasites during inspection and underline the importance of a correct consumers’ education.

## 1. Introduction

Over two-hundred different parasitic species of a variety of taxa are reported in the literature for cephalopods [1,2], mainly as larval and post-larval stages [3]. Although the interest of the scientific community on parasites associated with cephalopods is growing [4], the knowledge on specific aspects, including some parasites’ life cycle and transmission pathway, is still scarce [2]. 

The cephalopod class includes three worldwide appreciated commercial categories: squid (Myopsida and Oegopsida taxa), octopus (Octopoda) and cuttlefish (Sepiida). Their nutrient composition and the continuously growing worldwide popularity of raw seafood has prompted their demand increase [5]. Spain, Italy and Japan are the main consumers and importers of this kind of seafood [6]. Squid and octopus are particularly requested [5] and their tight supplies [7] are causing a strong rise in trade prices [6,7]. Therefore, products should comply with high-quality and hygienic standards to meet consumers’ requests and expectations. Therefore, Food Business Operators (FBOs) have to perform regular checks to avoid the commercialization of seafood obviously contaminated by visible parasites that are unfit for human consumption [8,9,10]. 

Species of squid, cuttlefish and octopus may act as intermediate or paratenic hosts in the life cycle of cestodes that mature in elasmobranchs and are transferred from host to host through the food chain [2,3]. According to the available literature, different species of cestodes, mainly belonging to the orders Phyllobothriidea, Tetraphyllidea and Trypanorhyncha, were detected as larval stage in almost all the commercial cephalopod species [1] (Table 1). These cestodes represent visible parasites—“*a parasite or a group of parasites which has dimension, colour or texture which is clearly distinguishable from fish tissues and can be seen without optical means of magnifying and under good light conditions for human*” according to the definition given by [11]. Visible parasites can represent a hazard or a defect depending on their potential zoonotic role and thus require the implementation of specific management measures along the supply chain to reduce their impact on consumer’s health and satisfaction [12,13]. Cephalopod species reaching the market as whole and fresh, in which parasites can be found viable, may be particularly affected, considering that cestodes are commonly found in digestive tracts, buccal mass, mesentery and mantle cavity [1]. 

During a larger survey on parasitic nematodes in selected species of fresh whole cephalopods [14], visible alive plerocercoids were visually detected in the viscera of some specimens of longfin inshore squid (*Doryteuthis pealeii*). The present study represents the first molecular record of *Clistobothrium* sp. larvae in *D. pealeii*, based on the analysis of DNA fragments from the variable D2 region of the large subunit (LSU) rRNA gene and from the cytochrome *c* oxidase subunit I (*COI*) gene. In addition to update some aspects of the epidemiology of the detected cestode, this work wants to discuss the impact on cephalopods’ quality, considering that *D. pealeii* is among the cephalopod species most commercialized as fresh and whole on the Italian market. 

## 2. Results and discussion

### 2.1. Morphological Identification

**Figure 1:**
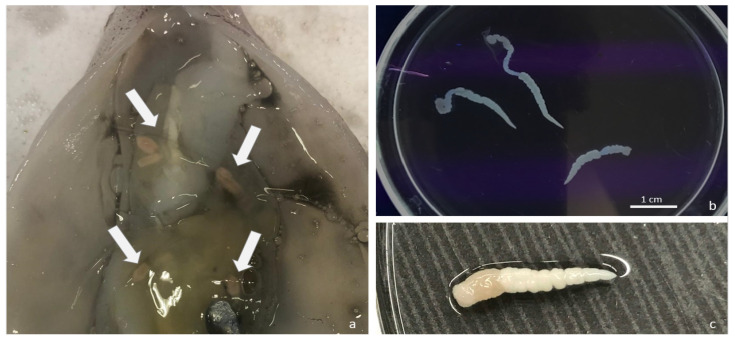
(**a**) Macroscopic aspect of the plerocercoid larvae at squid dissection, which were 1.5–3.5 cm long, whitish and actively mobile; (**b, c**) isolated larvae.

**Figure 2:**
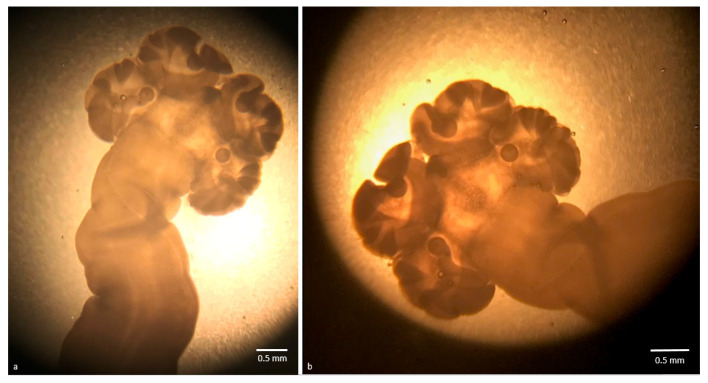
(**a**) Microscopic aspect of a plerocercoid larvae scolex, showing four large bothridia with folded margins, each with a rounded accessory sucker; (**b**) the same microscopic aspect of another plerocercoid.

In this work, a total of 18 alive plerocercoids were found in the viscera of 10 specimens of longfin inshore squid (P: 14.3%, 95% CI 7.1–24.7; MI: 1.8, MA: 0.26; range 1–4). Overall, *D. pealeii* specimens had a mean weight of 106.5 g (standard deviation, sd 29.1), a mean total length of 41.2 cm (sd 5.4) and a mean dorsal mantle length of 16.8 (sd 3.5). Details of the size of the positive squids are given in Table 2. A positive correlation was observed between the total weight and the number of parasites per host (r_s_ = 0.54, p (2-tailed) = 0.002), while no statistically significant correlation was found for the dorsal mantle length and the weight. Plerocercoids were 1.5–3.5 cm long, whitish and actively mobile (Figure 1, Video S1). Under optical microscopy the larvae presented an unarmed evaginated scolex, attached to a fusiform larval body, with an apical sucker surrounded by four large bothridia with folded margins, each showing a rounded accessory sucker (Figure 2). The observed characteristics allowed to identify the parasites as a “tetraphyllidean” (or phyllobothridean according to [25]) plerocercoid larvae. This kind of larvae, which should be referred to as Type XV [26], have historically been defined as *Phyllobothrium delphini*, but molecular sequence data have suggested that they may actually belong to *Clistobothrium* sp. [26]. Analogous larval types had been described in squids [27], teleosts [28] and in deepwater sharks [29]. However, considering the morphological uniformity of cestode larvae [3] and the renowned difficulty to reliably identify them [26,30,31], a morphological identification at species level was not achieved, and parasites were submitted to molecular analysis.

### 2.2. Molecular Analysis

#### 2.2.1. Molecular Target Selection

The LSU was selected as elective target for the analysis, as it is the most used molecular marker for identifying Cestoda [3,26,32,33,34,35,36,37,38], sometimes in combination with the small subunit (SSU) rRNA gene [39,40,41]. Although Olson et al. [40] indicated that the D2 region of the LSU gene exhibited sufficient variability to be useful for species-level identification, a low interspecies variability has been reported by other authors [35], and other genes, such as *COI* and ITS, were proposed as alternative markers for distinguishing closely related species of Phyllobothriidae [35,42,43]. Thus, the *COI* was used as additional target to better assess inter and intra-specific variability. However, reference sequences for this target region are scarce [35], as it can be observed in Table S1, where 427 sequences were retrieved from databases for the LSU, while only 72 sequences were available for the *COI* gene. A higher taxonomic coverage of LSU in comparison with *COI* is factually evident (Table S1).

#### 2.2.2. Large Subunit (LSU) rRNA Gene Analysis

LSU sequences were obtained from all the eighteen plerocercoid found. The BLAST analysis showed high similarity (> 99%) with sequences deposited as *Clistobothrium* cf. *montaukensis*, *Clistobothrium* sp., *C. montaukensis*, *Pelichnibothrium speciosum*, and with one record deposited as Tetraphyllidea sp. (KT148970), thus not allowing a specific identification. However, a 100% identity value was observed only with 6 sequences deposited as *Clistobothrium* cf. *montaukensis*, one deriving from an adult specimen isolated from *Lamna nasus* (JF436969) [36] and the remaining from plerocercoids found in the Patagonian squid *Doryteuthis gahi* (AF382071-72, AF382074, AF382079, AF382081) [3], although with a low query coverage (81–91%). Initially, both the LSU Neighbor Joining (NJ) and Maximum Likelihood (ML) phylograms were constructed with the complete dataset created as described in Section 3.4.2. Then, redundant sequences were removed, except for the genus *Clistobothrium*, for which all the available sequences have been included. Until recently, only three species were reported in the genus *Clistobothrium*: *C. montaukensis*, *C. carcharodoni* and *C. tumidum* [44]. However, Caira et al. [34] have very recently described two new species (*C. amyae* and *C. gabywalterorum*) and suggested an expansion of the total number to six, including the undescribed species *C.* cf. *montaukensis* reported by Brickle et al. [3] and Randhawa and Brickle [36].

**Figure 3:**
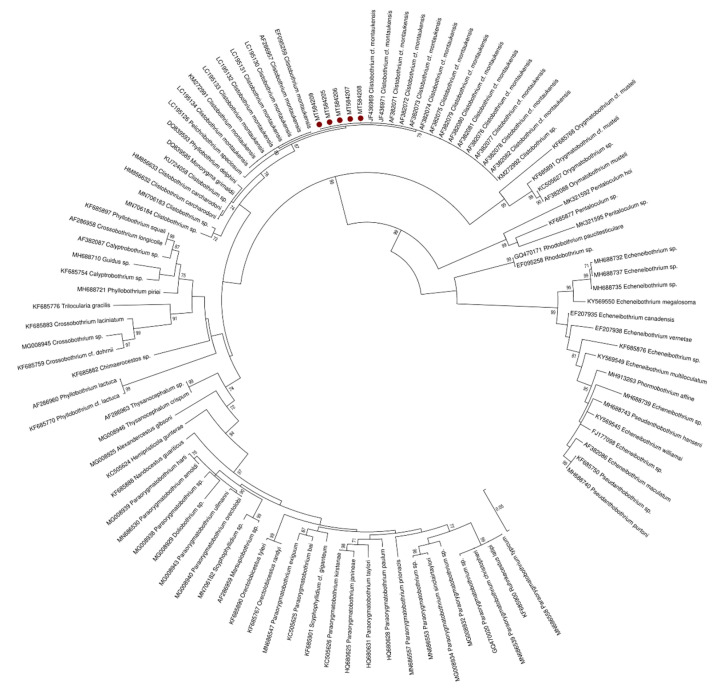
Maximum likelihood phylogram created with the large subunit (LSU) sequences of the species belonging to the Phyllobothriidae family retrieved from GenBank, together with 5 of those produced in this study. Redundant sequences were removed.

In both NJ and ML phylograms (Figure 3, only ML shown), the sequences from the plerocercoid larvae produced in this study clustered with the sequences deposited as *C.* cf. *montaukensis,* which retrieved 100% identity in the BLAST analysis, as well as with one sequence deposited as *Clistobothrium* sp. obtained from a cestode larva found in the oarfish *Regalecus russelii* [45] with a bootstrap value of 81. This cluster was separated from other *Clistobothrium* species, in particular *C. carcharodoni*, *C. montaukensis* and sequences of two newly described species of *Clistobothrium* sp. (*C. amyae* and *C. gabywalterorum*) [34], as well as from the other Phyllobothriidae species which showed an identity value higher than 99% in the BLAST analysis. Thus, the larvae found in the present study can be assigned to the genus *Clistobothrium,* possibly to the same undescribed species reported by Brickle et al. [3] and by Randhawa and Brickle [36] or maybe to another still undescribed species.

Several records of this genus without species level identification have been reported. Interestingly, Pardo Gandarillas et al. [17] found an unidentified Tetraphyllidea plerocercoid from the jumbo flying squid *Dosidicus gigas* morphologically very similar to the larvae found in this study and stated that the presence of an apical sucker-like structure, accessory sucker on each bothria and the folded and curled bothrial shape resembled *Phyllobothrium tumidum* (former name of *C. tumidum*) described by Stunkard [31]. In the work of Klotz et al. [35], genetically identified *Clistobothrium* sp. merocercoids were found in seals, and tentatively attributed to *C. tumidum* on the basis of bothridial morphology. However, further molecular analysis, ideally investigating also adult specimens, would be needed [35], also considering that the taxonomy of tetraphyllidean and phyllobothridean has undergone major revision [34,38,46]. In fact, the Phyllobothriidae family, which was traditionally included in the Tetraphyllidea order, was recently elevated to ordinal status [25,30]. 

#### 2.2.3. Cytochrome *c* Oxidase Subunit I (*COI*) Gene Analysis

**Figure 4:**
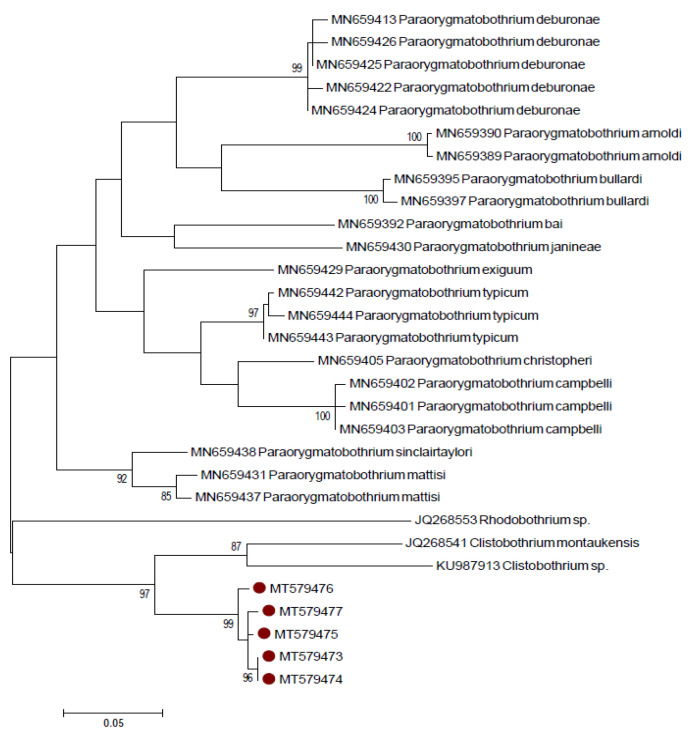
Maximum likelihood phylogram created with the Cytochrome *c* Oxidase Subunit I (*COI*) sequences (fragment A) of the species belonging to the Phyllobothriidae family retrieved from GenBank, together with 5 of those produced in this study. Redundant sequences were removed.

**Figure 5:**
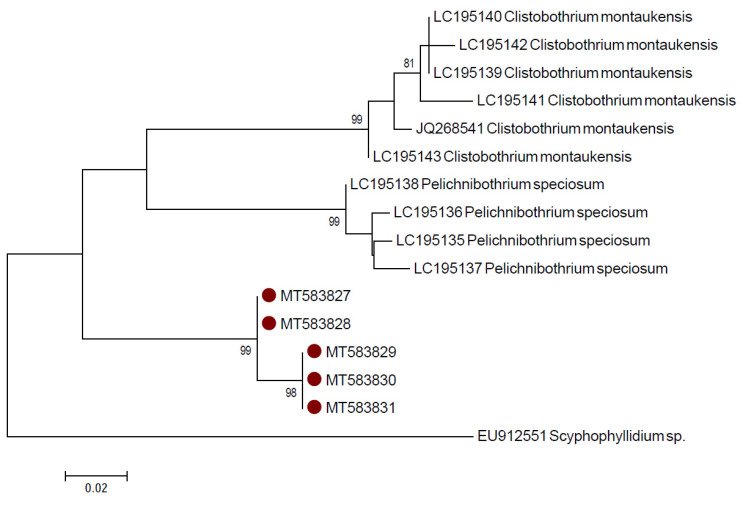
Maximum likelihood phylogram created with the *COI* sequences (fragment B) of the species belonging to the Phyllobothriidae family retrieved from GenBank, together with 5 of those produced in this study.

As already reported, the *COI* gene was used as an additional target to better assess inter-specific sequence variation. As observable in Table S1, a higher number of sequences is available for fragment A (n = 61) respect to fragment B (n = 11). However, fragment B was amplified to allow the comparison of our sequences with additional *Clistobothrium* sp. sequences, considering that most of the 11 fragment B sequences (n = 7) belong to this genus. The BLAST analysis conducted using the fragment A of the *COI* gene retrieved the highest percentage of identity (85.39–84.32%) with sequences of *C. montaukensis*, *Clistobothrium* sp., *Paraorygmatobothrium exiguum*, *P. typicum*, *P. christopheri* and *Rhinebothroides scorzai*, while the BLAST analysis with the fragment B of the *COI* gene showed the highest percentage of identity (87–88%) with sequences of *C. montaukensis* (AN: JQ268541, LC195139, LC195141–43) and also with *Pelichnibothrium speciosum* (LC195135–38). These results confirm the hypothesis based on the analysis of the LSU gene, also demonstrating that the larvae found in this study are not *C. montaukensis*. Similarly, no species-specific identification was achieved on BOLD: no match was obtained for both fragments comparing them with the Species Level Barcode Record database, while the comparison with the All Barcode Record database retrieved a highest match of fragment A with *C. montaukensis* (84.85–85.42%) and with *Schyzocotyle nayarensis* (82.31–83.08%). The BLAST and BOLD results for the *COI* gene should be interpreted taking into account the low number of available sequences for Phyllobothriidae and the inter-specific variability. In fact, the results of the pairwise distance analysis on fragment A showed a high inter-specific variability among species of the genus *Clistobothrium* (16.6–19.3%) (Table S2), and among species of the genus *Paraorygmatobothrium* (8.5–21.3%), the only genera of the Phyllobothriidae family for which sequences from more than one species were available. Similarly, also the difference between our sequences and *C. montaukensis* for fragment B was relatively high (13.8–16.7%) (data not shown). A similar inter-specific distance was already observed for the *COI* gene for *Clistobothrium* spp. [35] and for *Paraorygmatobothrium* spp. [38]. Finally, in both the NJ and ML phylograms of the fragment A of the *COI* gene, the sequences produced in this study appeared phylogenetically closer to the clade comprising the sequences from *C. montaukensis* (JQ268541) and *Clistobothrium* sp. (KU987913), although they clustered separately with a bootstrap value of 97 (Figure 4, only ML shown). The NJ and ML phylogram of fragment B confirm that our larval specimens belong to the genus *Clistobothrium* (Figure 5, only ML shown). In general, the low number of sequences and species available for the *COI* gene does not allow to achieve a species level identification but supports the LSU results. 

### 2.3. Viable and Visible Larval Cestodes of Clistobothrium sp: Epidemiology and Implication for Cephalopod Inspection

Tapeworms are ubiquitous residents of the spiral intestine of elasmobranchs, but their life cycles are poorly known. It is generally thought that two or three different intermediate host species are involved before the definitive host infection. Studies are severely hampered by the difficulties associated with identifying cestode larvae [26,30]. Applications of molecular methods have improved the situation, even though the paucity of molecular data for most adult marine tapeworms greatly limits this approach [26,36]. 

Although the complete life cycle of the species of the genus *Clistobothrium* is still unclear, the available data support the cycle exhaustively illustrated [35], with sharks as definitive hosts, crustaceans as 1st intermediate hosts of the procercoid larvae, bony fish/cephalopods/sea turtles as 2nd intermediate hosts of plerocercoid larvae and cetacean/pinnipeds as 3rd intermediate hosts of merocercoid larvae. Reliable host reports indicate that species of *Clistobothrium* are restricted to sharks of the Lamnidae family [44] as definitive hosts. In fact, the adult form of *C. carcharodoni* was described by [47] in the spiral intestine of the great white shark (*Carcharodon carcharias*). The great white shark was also identified as definitive host of *C. tumidum*, originally described as *Phyllobothrium tumidum*, and transferred to the genus *Clistobothrium* by [44]. The same author also described for the first time the species *C. montaukensis* from the spiral intestine of the shortfin mako shark (*Isurus oxyrinchus*) [44]. Subsequent studies confirmed the occurrence of *C. montaukensis* in shortfin mako [48] and of *C. carcharodoni* in the great white shark [37], while, as mentioned, a specific identification could not be achieved for the specimens identified as *C.* cf. *montaukensis* found in the porbeagle *Lamna nasus* [36]. A survey of deeper water sharks from the Azores found tetraphyllidean larval morphologically attributed to *Clistobothrium* sp. also in the birdbeak dogfish (*Deania calcea*) and the longnose velvet dogfish (*Centroselachus crepidater*) [29]. As regards 3rd intermediate hosts, subcutaneous merocercoids were recently found in two Cape fur seals (*Arctocephalus pusillus pusillus*) [35]. Although cephalopods are described as hosts of this genus, *Clistobothrium* sp. has not been commonly reported in the most recent parasitological studies (Table 1). In particular, Brickle et al. [3] examined the congeneric longfin Patagonian squid (*Doryteuthis gahi*) finding plerocercoids provisionally morphologically identified as *Phyllobothrium* sp. and attributed to *Clistobothrium* sp. after molecular analysis. The related sequences (AF382071-82) have been deposited as *Clistobothrium* cf. *montaukensis*. Interestingly, heavy infections with metacestodes named as *Phyllobothrium longilinis* have also been reported for *D. pealeii* [31]. Brickle et al. [3] also suggest that previous reports of plerocercoids of *Phyllobothrium* sp. in squid may have been in error, and that identifications have been further complicated by the historical use of the genus *Phyllobothrium* for all non-hooked tetraphyllidean worms with “leaf-like”, marginally crenulated bothridia. The use of the term *Phyllobothrium* with a broad sense can also be observed in some of the studies reported in Table 1. Thus, the presence of the genus *Clistobothrium* might have been underestimated. The current report represents the first molecular description of *Clistobothrium* sp. in *D. pealeii*, confirming the role of squids of the genus *Doryteuthis* as 2nd intermediate hosts [3,31].

The longfin inshore squid *D. pealeii* is a high valued species originating from the North West Atlantic, where its commercial catches started in the late 1800s. Still nowadays, *D. pealeii* is both sold internally and, to a lesser extent, exported. In particular, between 1991 and 2012 Italy was the first importer of *D. pealeii*, accounting for 29% of the exports [49]. 

*D. pealeii*, which is available as whole and fresh on the Italian market usually between May and June, is increasingly appreciated as an alternative for local squid species (authors’ personal communication). At the European level, fishery products must comply with the EU hygiene standards, based on the principles provided by the EC General Food Law [8]. As regards the presence of visible parasites, their possible effect on the quality of the product shall also be taken into consideration [15], and the Regulation (EC) No 853/2004 [10] states that sea-food products that are obviously contaminated with parasites should not be released for human consumption. The visual inspection has become the official method to be included within self-control programs for detecting visible parasites before market release and ensuring seafood quality and safety. In fact, beside the risk posed by zoonotic parasites, visually “un-aesthetic” parasites may decrease the seafood commercial value [50,51,52]. This might be the case of the visible and alive parasites described in this work. In fact, in case of products eviscerated at home by consumers, these “disgusting” larvae may become clearly visible to the naked eye. Thus, consumer education concerning the possibility that, despite FBOs and Official Veterinarians’ efforts, parasites might be present in wild seafood and information on correctly managing such defects should always be sought. This is in order to avoid excessive and unnecessary alarmism, which may also have negative media impact. 

## 3. Materials and Methods 

### 3.1. Squid Sampling

Overall, 70 *D. pealeii* (superorder Decapodiformes, order Teuthida, family Loliginidae, former name *Loligo pealeii*) specimens morphologically identified by experts according to the FAO morphological keys (http://www.fao.org/3/ac479e/ac479e00.htm) from the Northwest Atlantic (FAO area 21) were collected as whole fresh at the Wholesale fish market of Milan (n = 49) and at the distribution platforms of two leading brands in the organized distribution (n = 21) in June 2019. Three-four specimens were collected in each different sampling day and, overall, the specimens derived from 20 different batches. The squids collected at the Wholesale fish market of Milan were immediately submitted to visual inspection; the parasites were collected and stored separately; then, squids were frozen and transferred to the FishLab for further analysis. Squids collected at the platforms were instead directly transferred on ice to the FishLab where they were visually examined as fresh. 

### 3.2. Parasite Detection

Each squid specimen was measured, registering the total length (TL) and the dorsal mantle length (DML), and weighted (total weight-TW, viscera weight-VW, mantle weight-MW) before visual inspection. The squids were opened longitudinally on their ventral side and a visual inspection under natural light was performed according to Commission Regulation (CE) n. 2074/2005 on both the visceral mass (comprising the digestive, excretory and reproductive organs) and mantle of fresh specimens to detect visible parasites. The plerocercoid larvae were counted and washed in 0.9% NaCl solution (Pero, Milano, Italy). After microscopic observations of the key morphological features [26,27,28,31,53,54], they were preserved in 70% ethanol (Carlo Erba Reagents s.r.l., Barcelona, Spain) and stored at −20 °C until molecular identification. The Spearman correlation coefficient (*rho*) was used to assess the correlation between the TW, the DML, the TL of the cephalopod specimens and the number of parasites per host.

### 3.3. DNA Extraction and Evaluation

Total DNA extraction was performed from all the collected plerocercoid larvae, according to [55]. DNA concentration and purity were determined by a NanoDrop ND-1000 spectrophotometer (NanoDrop Technologies, Wilmington, Delaware, DE, USA).

### 3.4. Large Subunit (LSU) rRNA Gene Analysis

#### 3.4.1. PCR Amplification, Sequencing and Sequences Editing

A 780 bp fragment of the variable D2 region of the large subunit (LSU) rRNA gene was selected as elective target and amplified from all the 18 plerocercoid larvae with the primer pair TrypFOR1 (5’-AGTCGGGTTGTTTGAGAATG-3’) and TrypREV (5’-CGTGTTTCAAGACGGGTC-3’), routinely used in FishLab for cestode species identification. PCR amplifications were set up in a 20 μL reaction volume containing 2 μL of a 10× buffer (Biotechrabbit GmbH, Hennigsdorf, Germany), 200 μM of each dNTP (dNTPmix, EurocloneS.p.A-Life Sciences Division, Pavia, Italy), 250 nM of each primer, 2.5 U PerfectTaq DNA Polymerase (Biotechrabbit GmbH, Hennigsdorf, Germany), 50–100 ng of DNA and DNase free water (Water Mol. Bio. Grade, DNase-RNase and Protease free, 5Prime GmbH, Hamburg, Germany). The following cycling program was used: initial denaturation at 95 °C for 3 min; 35 cycles at 95 °C for 25 s, 50 °C for 25 s, 72 °C for 35 s; final extension at 72 °C for 5 min. PCR products were analyzed by electrophoresis in 2% agarose gel, and amplicons were subsequently sent for standard forward and reverse Sanger sequencing to an external company. The obtained sequences were analyzed, edited and assembled with the Geneious R7 software (Biomatters Ltd, Auckland, New Zealand) [56]. Fine adjustments were manually made after visual inspection. Five sequences representative of the haplotypes were deposited in GenBank (accession numbers MT584205–MT584209).

#### 3.4.2. Comparison with Databases and Phylogenetic Analysis

The edited sequences were used to run a BLAST analysis on GenBank, selecting the Somewhat similar sequences (blastn) algorithm. Then, genera of the Phyllobothridae family were searched on the World Register of Marine Species database [57] and also in the most recent works dealing with Phyllobothriidea taxonomy [25,34,38]. Subsequently, for all the retrieved genera, all the available LSU sequences were searched on GenBank (https://www.ncbi.nlm.nih.gov/genbank/) to create a genetic dataset, as detailed in Table S1. Both valid and synonym genus names, as well as *taxa inquirenda*, were used, to make the collection as exhaustive as possible. All the retrieved sequences, together with those produced in this study, were then aligned with Geneious R7 software (Biomatters Ltd, Auckland, New Zealand) [56] and a Neighbor-Joining (NJ) and Maximum Likelihood (ML) phylograms were constructed using the Kimura 2-parameter model [58] with 1000 bootstrap re-samplings in MEGA-X [59].

### 3.5. Cytochrome c Oxidase Subunit I (COI) Gene Analysis

#### 3.5.1. Primers Projecting

**Figure 6:**
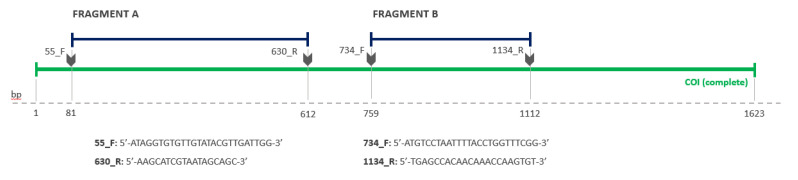
Cytochrome *c* oxidase subunit I *(COI)* gene primers projected in this study: fragment A and B position in relation to the complete *COI* complete gene and primers’ sequences.

Primers for the *COI* gene were *ex novo* projected in this study by using the Geneious R7 software (Biomatters Ltd, Auckland, New Zealand) [56]. In order to do so, all the available *COI* sequences from the Phyllobothriidae family, collected as described in Section 3.4.2, were retrieved from GenBank (https://www.ncbi.nlm.nih.gov/genbank/) database (see Table S1 for details), and aligned with the Geneious R7 software (Biomatters Lts, Auckland, New Zealand) [56]. After the alignment, two different groups of sequences were observed, corresponding to two distinct regions of the COI gene. Thus, two primer pairs, 55_F/630_R and 734_F/1134_R, were projected for amplifying fragments of 532 bp (fragment A) and 354 bp (fragment B), respectively, from each region (Figure 6).

#### 3.5.2. PCR Amplification, Sequencing and Sequences Editing

PCR amplifications were set up in a 20 μL reaction volume as described in Section 3.4.1 with the following cycling programs: initial denaturation at 95 °C for 3 min; 40 cycles at 95 °C for 25 s, 48 °C (fragment A)/ 54 °C (fragment B) for 25 s, 72 °C for 30 s; final extension at 72 °C for 5 min. Fragment A and B PCR products were visualized and sequenced as described in Section 3.4.1. The obtained sequences were analyzed, edited and assembled with the Geneious R7 software (Biomatters Lts, Auckland, New Zealand) [56]. Five representative sequences per fragment were deposited in GenBank (accession numbers: MT579473–MT579477 fragment A; MT583827–MT583831 fragment B).

#### 3.5.3. Comparison with Databases, and Phylogenetic Analysis

For both fragment A and B, the BLAST analysis on GenBank was conducted as described for the LSU (Section 3.4.2) and the Identification System (IDs) on BOLD was also used. In addition, a pairwise distance matrix by the use of p-distance model with 1000 nonparametric bootstrap replicates was produced using MEGA-X. NJ and ML phylograms were constructed for both fragments using the datasets obtained by database sequences collection (see Section 3.4.2, including the same sequences used for the primer projecting (Section 3.5.1).

## 4. Conclusions

The life cycles of marine cestodes, especially those maturing in sharks and rays, are poorly known, mainly due to difficulties in larval stages identification [2,26]. Issues have already been highlighted within the Phyllobothriidea order, for example, where the use of molecular methods has often been advocated [32,35]. To our knowledge, in this work the presence of molecularly identified plerocercoid larvae of *Clistobothrium* sp. in longfin inshore squid (*D. pealeii*) was assessed for the first time. The results contribute to further elucidate the life cycle of this parasite. Beside an epidemiological relevance, FBOs and official authorities should be aware of the possible presence of live visible plerocercoid larvae in fresh longfin inshore squid sold as fresh on the market. Although not presenting a public health risk, these may present defects affecting cephalopods, constituting a reason of disgust and loss of trust in the control systems for consumers. Thus, consumer education to avoid excessive and unnecessary alarmism is important, particularly for cephalopods sold fresh and whole such as *D. pealeii* that may contain visible parasites still viable as reported in this study. 

## Figures and Tables

**Table 1:** Overview of other studies available in the literature (1991–2020) reporting cestodes in cephalopods.

Ref.	Samp. Period	Geographical Area	Cephalopod Common Name (Scientific Name), n of Examined Specimens	Species (Family, Order)	Localization	P (%)	Parasite ID
Guillén-Hernández et al., (2018) [4]	August, 2009–June, 2010	Yucatán Peninsula, Mexico (FAO 31)	Mexican four-eyed octopus (*Octopus maya*), 1202	*Prochristianella* sp. (Eutetrarhynchidae, Trypanorhynca)	buccal mass, oesophagus, cecum, intestine	57.0–98.0	Morph.
				*Eutetrarhynchus* sp. (Eutetrarhynchidae, Trypanorhynca)	digestive gland, esophagus, intestine, ink sac	7.0–59.1	
				*Nybelinia* sp. (Tentaculariidae, Trypanorhynca)	buccal mass, esophagus, intestine	0.4–51.2	
				*Echeneibothrium* sp. (Echeinebothriidae, Rhinebothriidea)	cecum, intestine	4.0–21.0	
				*Prosobothrium* sp. (Prosobothriidea, Onchoproteocephalidea)	digestive gland, ink sac	16.8–27.0	
				Tetraphyllidea	cecum, intestine	1.0–7.0	
				Unidentified plerocercoid	digestive gland, ink sac, gills	10–26.6	
Cavaleiro, (2013) [15]	2010	Matosinhos, Portugal, NE Atlantic (FAO 27)	common octopus (*Octopus vulgaris*), 120	*Nybelinia* sp. (Tentaculariidae, Trypanorhynca)	stomach, intestine	4.2	Morph.
Petrić et al., (2011) [16]	October, 2007–October, 2008	Central Adriatic Sea (FAO 37.2.1)	shortfin squid (*Illex coindetti*), 439	*Phyllobothrium* sp. (Phyllobothriidae, Phyllobothriidea)	stomach	2.3	Morph.
Pardo-Gandarillas et al., (2009) [17]	July, 2003– February, 2004	Central-Southern Chile (FAO 87)	jumbo flying squid (*Dosidicus gigas*), 124	*Hepatoxylon trichiuri* (Sphyriocephalidae, Trypanorhynca)	mantle cavity, gonads, stomach	70.2	Morph.
				*Tentacularia coryphaenae* (Tentaculariidae, Trypanorhynca)	mantle cavity, gonads	5.6	
				Plerocercoid larvae (Tetraphyllidea)	stomach, cecum and intestine	83.1	
				*Pelichnibothrium speciosum* (Phyllobothriidae, Phyllobothriidea)	intestine	NR	
Nigmatullin et al., (2009) [18]	1981–1984	south part of the eastern Pacific (FAO 87)	neon flying squid (*Ommastrephes bartramii*),60	*Tentacularia coryphaenae* (Tentaculariidae, Trypanorhynca)	whole mantle cavity	9.1	Morph.
				*Scyphophyllidium* sp. (Phyllobothriidae, Phyllobothriidea)	stomach cavity and cecum	4.5	
Brickle et al., (2001) [3]	February, 1999–June, 2000	Falkland Islands (South Atlantic Ocean) (FAO 41.3.2)	longfin Patagonian squid (*Doryteuthis gahi*), 1096	*Clistobothrium montaukensis* (Phyllobothriidae, Phyllobothriidea)	cecum, intestine, stomach, mantle	5.75	Molec.
				*Ceratobothrium xanthocephalum* (Gastrolecithidae, Tetraphyllidea)			
Shukhgalter and Nigmatullin, (2001) [19]	1981–1989	East Pacific Ocean (FAO 77 and 87)	jumbo squid (*Dosidicus gigas*), 849	*Pelichnibothrium speciosum* (Phyllobothriidae, Phyllobothriidea)	rectum, cecum, stomach	75.2	Morph.
				*Phyllobothrium* sp. (Phyllobothriidae, Phyllobothriidea)	rectum, cecum, stomach, mantle cavity, buccal cone	1.2	
				*Tentacularia coryphaenae* (Tentaculariidae, Trypanorhynca)	mantle cavity, mantle	6.6	
Gonzalez and Kroeck, (2000) [20]	July–November, 1993	South West Atlantic St. Matias gulf (FAO 41.3.1)	Argentine short-fin squid (*Illex argentinus*), 91	*Prosobothrium* sp. (Prosobothriidea, Onchoproteocephalidea)	viscera	100.0	Morph.
				Onchobotriidae (Onchoproteocephalidea)		0.0–100.0	
				*Nybelinia linguaris* (Tentaculariidae, Trypanorhynca)		0.0–21.0	
Gestal et al., (1998) [21]	December, 1994–December, 1995	Galician coast, Spain (FAO area 27.9)	Common octopus (*Octopus vulgaris*), 100	*Phyllobothrium* sp. (Phyllobothriidae, Phyllobothriidea)	Intestine, cecum	3.0	Morph.
				*Nybelinia lingualis* (Tentaculariidae, Trypanorhynca)	Mouth, stomach, cecum	3.0	
Pascual et al., (1996) [22]	1992–1995	coast of Galicia, Spain (FAO 27.9)	broadtailed short-fin squid (*Illex coindetti*), 600	*Phyllobothrium* sp. (Phyllobothriidae, Phyllobothriidea)	NR	48.0	Morph.
				*Pelichnibothrium speciosum* (Phyllobothriidae, Phyllobothriidea)		0.3	
				*Dinobothrium* sp. (Gastrolecithidae, Tetraphyllidea)		1.0	
				*Nybelinia yamagutii* (Tentaculariidae, Trypanorhynca)		0.7	
			European squid (*Loligo vulgaris*), 8	*Phyllobothrium* sp. (Phyllobothriidae, Phyllobothriidea)		62.5	
			European flying squid (*Todarodes sagittatus*), 65	*Phyllobothrium* sp. (Phyllobothriidae, Phyllobothriidea)		20.0	
			lesser flying squid (*Todaropsis eblanae*), 600	*Phyllobothrium* sp. (Phyllobothriidae, Phyllobothriidea)		31.2	
				*Pelichnibothrium speciosum* (Phyllobothriidae, Phyllobothriidea)		0.7	
				*Nybelinia linguaris* (Tentaculariidae, Trypanorhynca)		0.3	
			common cuttlefish (*Sepia officinalis*), 38	*Phyllobothrium* sp. (Phyllobothriidae, Phyllobothriidea)		2.6	
			pink cuttlefish (*Sepia orbignyana*), 22	*Phyllobothrium* sp. (Phyllobothriidae, Phyllobothriidea)		9.0	
			common octopus (*Octopus vulgaris*), 70	*Phyllobothrium* sp. (Phyllobothriidae, Phyllobothriidea)		4.3	
			lesser octopus (*Eledone cirrhosa*), 67	*Phyllobothrium* sp. (Phyllobothriidae, Phyllobothriidea)		10.4	
Pascual et al., (1994) [23]	October, 1991–April, 1992	North Galician Shelf waters (FAO 27.8)	broadtailed short-fin squid (*Illex coindetti*), 70	*Phyllobothrium* sp. (Phyllobothriidae, Phyllobothriidea)	cecum, stomach	87	Morph.
				*Dinobothrium septaria* (Gastrolecithidae, Tetraphyllidea)			
Bower and Margolis (1991) [24]	Summer 1987	West coast of North America (FAO 21)	flying squid (*Ommastrephes bartramii*), 68	*Phyllobothrium* sp. (Phyllobothriidae, Phyllobothriidea)	esophagus, stomach, cecum, intestine, rectum, gills, gonads	94.1	Morph.
				*Tentacularia* sp. (Tentaculariidae, Trypanorhynca)	NR	1.5	
				*Rhadinorhynchus* sp. (Rhadinorynchidae, Echinorynchida)	NR	NR	

Ref: reference; Samp. period: Sampling period; P (%): prevalence expressed as percentage.

**Table 2:** Details of the weight and length of the positive longfin inshore squid (*Dorytheutis pealeii*) specimens and the number of larvae found in each specimen.

Host Sample Code	Total Weight	Total Length	Mantle Lengths	Viscera Weight	Mantle Weight	N Plerocercoid Larvae
D. PEA-23	117	57	22	8	109	1
D. PEA-30	87	36	12	18	45	1
D. PEA-33	141	41	12	6	92	2
D. PEA-34	136	44	12	20	70	1
D. PEA-35	153	52	23	26	94	1
D. PEA-45	175	43	15	15	79	2
D. PEA-58	119	36	24	6	73	3
D. PEA-63	163	48	24	17	97	1
D. PEA-65	152	46	23	13	89	4
D. PEA-67	136	44	22	13	83	2
**Overall**						**18**
**Mean (ds)**	**137.9 (25.5)**	**44.7 (6.5)**	**18.9 (5.4)**	**14.2 (6.4)**	**83.1 (17.8)**	

## Supplementary Materials

The following are available online at https://www.mdpi.com/2076-0817/9/7/596/s1. Table S1: Genetic dataset including all the available sequences of the large subunit (LSU) rRNA and cytochrome *c* oxidase subunit I (*COI*) genes of the Phyllobothridae family. Table S2: Average cytochrome *c* oxidase subunit I (*COI*) gene sequences divergences (fragment A). Video S1: Macroscopic aspect of the live plerocercoid larvae at squid dissection.
