# Supplementary material for: Molecular Characterization of Clistobothrium sp. Viable Plerocercoids in Fresh Longfin Inshore Squid (Doryteuthis pealeii) and Implications for Cephalopod Inspection
Source: Pathogens. 2020 Jul 21;9(7):596. doi: 10.3390/pathogens9070596 (PMC7400031; doi:10.3390/pathogens9070596)
Supplement: Supplementary file 1 [file pathogens-09-00596-s001.zip › Table S1.docx]

**Table S1.** Genetic dataset including all the available sequences of the large subunit (LSU) rRNA and cytochrome *c* oxidase subunit I (*COI*) genes of the Phyllobothridae family.

| **Genus** | **Species** | **LSU Sequences** | **Number LSU seq.** | **COI Sequences (Fragment A)** | **Number COI seq. (Fragment A)** | **COI Sequences (Fragment b)** | **Number COI seq. (Fragment B)** |
| --- | --- | --- | --- | --- | --- | --- | --- |
| *Alexandercestus* | *A. gibsoni* | MG008925  KC505623 | 2 | - |  | - |  |
| [*Bibursibothrium*](http://www.marinespecies.org/aphia.php?p=taxdetails&id=515882) |  | - |  | - |  | - |  |
| *Calyptrobothrium* | *Calyptrobothrium* sp. | KF685754  AF382087 | 2 | - |  | - |  |
| *C[ardiobothrium](http://www.marinespecies.org/aphia.php?p=taxdetails&id=515885)* |  | - |  | - |  | - |  |
| *Caulopatera* |  | - |  | - |  | - |  |
| *Chimaerocestos* | *Chimaerocestos* sp. | KF685882  KF685758 | 2 | - |  | - |  |
| *Clistobothrium* | *C.* [*carcharodoni*](https://www.ncbi.nlm.nih.gov/nuccore/HM856633.1) | HM856633  HM856632 | 2 | - |  | - |  |
|  | *C. mountakensis* | LC195130-34  KM272991  EF095259  AF286957 | 8 | JQ268541^a^ | 1 | LC195139-43  JQ268541^a^ | 6 |
|  | *C.* cf*. mountakensis* | JF436971  JF436969  AF382071-82 | 14 | - |  | - |  |
|  | *Clistobothrium* sp. | MN706183-84  KU724058  KM272992 | 4 | KU987913 | 1 | - |  |
| [*Crossobothrium*](https://www.ncbi.nlm.nih.gov/nuccore/KF685759.1) | *C.* cf. *dohrnii* | KF685759 | 1 | - |  | - |  |
|  | *C. laciniatum* | KF685883 | 1 | - |  | - |  |
|  | *C. longicolle* | AF286958 | 1 | - |  | - |  |
|  | [*Crossobothrium*](https://www.ncbi.nlm.nih.gov/nuccore/KF685759.1)sp. | MG008945 | 1 | - |  | - |  |
| *Discobothrium (invalid, accepted as Echeneibothrium)* |  | - |  | - |  | - |  |
| *Doliobothrium (invalid, accepted as Scyphophyllidium)* |  | MG008929.1  MG008928.1 | 2 | - |  | - |  |
| *Echeneibothrium* | *E. megalosoma* | KY569551  KY569550 | 2 | - |  | - |  |
|  | *E. multiloculatum* | KY569549  KY569548  KY569547  KY569546  MH688748 | 5 | - |  | - |  |
|  | *E. williamsi* | KY569545  KY569544  KY569543  MH688742 | 4 | - |  | - |  |
|  | *Echeneibothrium* sp. | MH924010  MH924009  MH924008  MH924007  MH924006  MH924005  MH924004  MH688747  MH688739  MH688738  MH688737  MH688736  MH688735  MH688734  MH688733  MH688732  MH688731  KF685876  FJ177098 | 19 | - |  | - |  |
|  | *E. canadensis* | MH688751  EF207848  EF207843  EF207842 | 4 | - |  | - |  |
|  | *E. vernetae* | MH688750.1  MH688749.1  EF207946.1  EF207945.1  EF207944.1  EF207943.1  EF207942.1  EF207941.1  EF207940.1  EF207939.1  EF207938.1  EF207937.1  EF207936.1  EF207935.1  EF207899.1  EF207898.1  EF207888.1  EF207887.1  EF207886.1  EF207885.1  EF207883.1  EF207881.1  EF207873.1  EF207872.1  EF207863.1  EF207862.1  EF207856.1  EF207855.1  EF207854.1  EF207852.1  EF207851.1  EF207847.1  EF207846.1  EF207845.1 | 34 | - |  | - |  |
|  | *E. maculatum* | MH688746  AF382086 | 2 | - |  | - |  |
| *Flexibothrium* |  | - |  | - |  | - |  |
| *Guidus* | *Guidus* sp. | MH688710 | 1 | - |  | - |  |
| [*Hemipristicola*](http://www.marinespecies.org/aphia.php?p=taxdetails&id=1036169) | *H.* [*gunterae*](https://www.ncbi.nlm.nih.gov/nuccore/KC505624.1) | KC505624  HQ680623  HQ680622  HQ680624  MN686526 | 5 | - |  | - |  |
| [*Monorygma*](https://www.ncbi.nlm.nih.gov/nuccore/DQ839586.1) | *M. grimaldii* | DQ839586  DQ839585  DQ839584  DQ839583  DQ839582  AY741598  AY741597  AY741596  AY741592  AY741591  AY741595  AY741594  AY741593 | 13 | - |  | - |  |
| *Marsupiobothrium (invalid, accepted as Scyphophyllidium)* | *Marsupiobothrium* sp | AF286959  KF685771 | 2 | - |  | - |  |
| *Nandocestus (invalid, accepted as Scyphophyllidium)* | *N. guariticus* | KF685888 | 1 | - |  | - |  |
| [*Orygmatobothrium*](https://www.ncbi.nlm.nih.gov/nuccore/KF685891.1) | *O.* cf. *musteli* | KF685891  KF685768 | 2 | - |  | - |  |
|  | *O. musteli* | AF382088 | 1 | - |  | - |  |
|  | [*Orygmatobothrium*](https://www.ncbi.nlm.nih.gov/nuccore/KF685891.1) *s*p. | KC505627 | 1 | - |  |  |  |
| *Orectolobicestus (invalid, accepted as Scyphophyllidium)* | *O. tyleri* | MG008930  KF685890 | 2 | - |  | - |  |
|  | *O. randyi* | KF685767 | 1 | - |  | - |  |
| *Paraorygmatobothrium* | *Paraorygmatobothrium* sp. | GQ470001-55  MN686551-55  MG008944  MG008938  MG008927  MG008926 | 64 | - |  | - |  |
|  | *P. typicum* | MN686558-60 | 3 | MN659439-45 | 7 | - |  |
|  | *P. prionacis* | MN686557  KF685892 | 2 | - |  | - |  |
|  | *P. paulum* | MN686556 | 1 | - |  | - |  |
|  | *P. janineae* | MN686550  HQ680625-27 | 4 | MN659430 | 1 | - |  |
|  | *P. exiguum* | MN686548  MN686547  KF685822  KF685769 | 4 | MN659429  MN659428 | 2 | - |  |
|  | *P. christopheri* | MN686539  MG008931 | 2 | MN659405  MN659404 | 2 | - |  |
|  | *P. arnoldi* | MN686527-30 | 4 | MN659388-90 | 3 | - |  |
|  | *P. sinclairtaylori* | MG008932-37 | 6 | MN659438. | 1 | - |  |
|  | *P. mattisi* | - |  | MN659431-37 | 7 | - |  |
|  | *P. deburonae* | - |  | MN659406-27 | 22 | - |  |
|  | *P. campbelli* | - |  | MN659400-04  MN659399 | 5 | - |  |
|  | *P. bullardi* | - |  | MN659393-98 | 6 | - |  |
|  | *P. bai* | KC505625 | 1 | MN659391-92 | 2 | - |  |
|  | *P. ullmanni* | MG008942-43 | 2 | - |  | - |  |
|  | *P. orectolobi* | MG008940-41 | 2 | - |  | - |  |
|  | *P. harti* | MG008939 | 1 | - |  | - |  |
|  | *P. kirstenae* | KC505626 | 1 | - |  | - |  |
|  | *P. taylori* | HQ680631-33 | 3 | - |  | - |  |
|  | *P. paulum* | HQ680628-30 | 3 | - |  | - |  |
| *Pelichnibothrium* | *P. speciosum* | LC195129  LC195128  LC195127  LC195126 | 4 | - |  | LC195135-38 | 4 |
| *Pentaloculum (taxon inquirendum)* | *P. hoi* | MK321594  MK321593  MK321592 | 3 | - |  | - |  |
|  | *Pentaloculum s*p. | KF685877  MK321595 | 2 | - |  | - |  |
| *Pillersium (taxon inquirendum)* |  | n.a. |  | - |  | - |  |
| *Phormobothrium* | *P. affine* | MH913272  MH913267  MH913263 | 3 | - |  | - |  |
| [*Phyllobothrium*](https://www.ncbi.nlm.nih.gov/nuccore/DQ839593.1) | *P. delphini* | DQ839593  DQ839592  DQ839591  DQ839590  DQ839589  JF506258  AY741606  AY741605  AY741604  AY741603  AY741602  AY741601  AY741599  AY741600 | 14 | - |  | - |  |
|  | *P. floraforme*^b^ | KY587528  MG372006 | 2 | - |  | - |  |
|  | *P. piriei* | MH688721 | 1 | - |  | - |  |
|  | *P. squali* | KF685897  KC543441 | 2 | - |  | - |  |
|  | *P.* cf. *lactuca* | KF685770  KC505628 | 2 | - |  | - |  |
|  | *P. lactuca* | AF286960 | 1 | - |  | - |  |
| *Pithophorus* |  | - |  | - |  | - |  |
| *Proboscidosaccus (invalid, accepted as Rhodobothrium)* |  | - |  | - |  | - |  |
| [*Pseudanthobothrium*](http://www.marinespecies.org/aphia.php?p=taxdetails&id=159476) | *P. hanseni* | MH688743-45  EF207910-34  EF207849-50  EF207844  EF207818-41 | 55 | - |  | - |  |
|  | *P. purtoni* | MH688741  MH688740  EF207909-00  EF207897-89  EF207882  EF207880-74  EF207871-64  EF207861-57  EF207853  EF207817-00  EF207799-88 | 73 | - |  | - |  |
|  | [*Pseudanthobothrium*](http://www.marinespecies.org/aphia.php?p=taxdetails&id=159476) *s*p. | KF685841  KF685750 | 2 | - |  | - |  |
| *Rhodobothrium* | *Rhodobothrium* sp. | FJ177100  JX088628  EF095258 | 3 | JQ268553.1 | 1 | - |  |
|  | *R. paucitesticolare* | GQ470171-78 | 8 | - |  | - |  |
| *Ruhnkecestus* | *latipi* | KF685900 | 1 | - |  | - |  |
| *Scyphophyllidium* | *Scyphophyllidium* sp. | MN706182 | 1 | - |  | EU912551 | 1 |
|  | *S.* cf. *giganteum* | KF685901 | 1 | - |  | - |  |
| *Thysanocephalum* | *T. crispum* | KF685902  MG008946 | 2 | - |  | - |  |
|  | *Thysanocephalum* sp. | AF286963 | 1 |  |  | - |  |
| *Tiarabothrium* |  | - |  | - |  | - |  |
| *Trilocularia* | *T. gracilis* | KF685776 | 1 |  |  | - |  |

^a^complete COI; ^b^the sequences were not included in the phylogram due to bad quality
