# Supplementary material for: Molecular Characterization of Clistobothrium sp. Viable Plerocercoids in Fresh Longfin Inshore Squid (Doryteuthis pealeii) and Implications for Cephalopod Inspection
Source: Pathogens. 2020 Jul 21;9(7):596. doi: 10.3390/pathogens9070596 (PMC7400031; doi:10.3390/pathogens9070596)
Supplement: Supplementary file 1 [file pathogens-09-00596-s001.zip › Table S2.docx]

Table S2. Average cytochrome *c* oxidase subunit I (*COI*) gene sequences divergences (fragment A).

|  |  | **1** | **2** | **3** | **4** | **5** | **6** | **7** | **8** | **9** | **10** | **11** | **12** | **13** | **14** | **15** |
| --- | --- | --- | --- | --- | --- | --- | --- | --- | --- | --- | --- | --- | --- | --- | --- | --- |
| 1 | Plerocercoid D.PEA | 0-1.7 |  |  |  |  |  |  |  |  |  |  |  |  |  |  |
| 2 | *C. montaukensis* (JQ268541) | 16.6-17.3 | - |  |  |  |  |  |  |  |  |  |  |  |  |  |
| 3 | *Clistobothrium sp.* (KU987913) | 18.6-19.3 | 17.4 | - |  |  |  |  |  |  |  |  |  |  |  |  |
| 4 | *P. arnoldi* (MN659388-90) | 23.0-24.5 | 22.4 | 27.9 | 0.0-0.5 |  |  |  |  |  |  |  |  |  |  |  |
| 5 | *P. bai* (MN659391-92) | 23.0-23.1 | 26.9 | 28.5 | 20.0-20.4 | 0.0 |  |  |  |  |  |  |  |  |  |  |
| 6 | *P. bullardi* (MN659393-98) | 24.3-26.2 | 22.0-22.4 | 24.5-25.3 | 19.2-19.9 | 19.7-20.4 | 0-1.4 |  |  |  |  |  |  |  |  |  |
| 7 | *P. campbelli* (MN659399-403) | 19.4-21.3 | 26.8-27.5 | 25.5-25.9 | 19.1-20.2 | 21.0-21-3 | 20.0-21.5 | 0.3-1.1 |  |  |  |  |  |  |  |  |
| 8 | *P. christopheri* (MN659404-05) | 18.1-18.5 | 24.9 | 24.6 | 19.4 | 19.2 | 19.3-19.4 | 9.4-10.0 | 0.0 |  |  |  |  |  |  |  |
| 9 | *P. deburonae* (MN659412-14; MN659421-27) | 20.3-22.5 | 24.2-25.0 | 25.2-26.0 | 16.8-18.2 | 17.3-18.6 | 15.8-16.8 | 15.8-17.5 | 15.2-16.2 | 0.0-1.9 |  |  |  |  |  |  |
| 10 | *P. exiguum* (MN659428-29) | 18.5-20.0 | 23.1-24-3 | 24.6-25.4 | 18.5-19.6 | 17.0-17.8 | 19.2-20.7 | 13.7-15.4 | 11.1-12.1 | 14.1-17.1 | 0-0.8 |  |  |  |  |  |
| 11 | *P. janineae* (MN659430) | 21.6-22.7 | 24.4 | 23.1 | 22.2-22.9 | 17.0 | 18.9-19.3 | 16.6-16.9 | 16.9 | 17.6-18.7 | 18.7-19.0 | 0.0 |  |  |  |  |
| 12 | *P. mattisi* (MN659431-37) | 19.2-21.4 | 25.3-26.1 | 24.0-25.1 | 19.4-19.9 | 18.1-18.9 | 18.2-19.2 | 13.8-15.9 | 12.8-14.8 | 14.2-16.3 | 16.0-18.5 | 19.4-21.3 | 0.0-1.9 |  |  |  |
| 13 | *P. sinclairteylori* (MN659438) | 20.5-21.6 | 25.4 | 24.9 | 20.2 | 16.9 | 18.7-19.4 | 16.5-17.2 | 14.8 | 16.6-17.7 | 18.1-19.2 | 19.5 | 5.7-6.3 | 0.0 |  |  |
| 14 | *P. typicum* (MN659439-45) | 17.6-19.8 | 24.1-24.9 | 21.8-23.0 | 20.2-21.3 | 18.6-19.7 | 16.1-17.1 | 10.0-10.9 | 8.5-9.4 | 12.7-14.8 | 12.7-14.4 | 15.8-16.1 | 11.7-15.1 | 13.0-14.1 | 0.0-1.4 |  |
| 15 | *Rhodobothrium* (JQ268553) | 32.4-34.2 | 35.3 | 33.0 | 29.5-29.9 | 29.1 | 30.6-31.4 | 29.1-29.5 | 30.2 | 27.6-29.1 | 30.0-30.5 | 30.2 | 27.5-28.6 | 25.1 | 28.0-28.8 | - |
